# Chinese Herbal Medicines for Rheumatoid Arthritis: Text-Mining the Classical Literature for Potentially Effective Natural Products

**DOI:** 10.1155/2020/7531967

**Published:** 2020-04-28

**Authors:** Xuan Xia, Brian H. May, Anthony Lin Zhang, Xinfeng Guo, Chuanjian Lu, Charlie C. Xue, Qingchun Huang

**Affiliations:** ^1^The Second Clinical College of Guangzhou University of Chinese Medicine, No. 111, Dade Road, Yuexiu District, Guangzhou 510120, China; ^2^Department of Rheumatology and Immunology, Guangdong Provincial Hospital of Chinese Medicine, No. 55, Neihuanxi Road, Higher Education Mega Center, Guangzhou 510006, China; ^3^China-Australia International Research Centre for Chinese Medicine, RMIT University, P.O. Box 71, Bundoora, Victoria 3083, Australia

## Abstract

**Background:**

Rheumatoid arthritis (RA) is an autoimmune disease characterized by multijoint swelling, pain, and destruction of the synovial joints. Treatments are available but new therapies are still required. One source of new therapies is natural products, including herbs used in traditional medicines. In China and neighbouring countries, natural products have been used throughout recorded history and are still in use for RA and its symptoms. This study used text-mining of a database of classical Chinese medical books to identify candidates for future clinical and experimental investigations of therapeutics for RA.

**Methods:**

The database Encyclopaedia of Traditional Chinese Medicine (*Zhong Hua Yi Dian*) includes the full texts of over 1,150 classical books. Eight traditional terms were searched. All citations were assessed for relevance to RA. *Results and Conclusions*. After removal of duplications, 3,174 citations were considered. After applying the exclusion and inclusion criteria, 548 citations of traditional formulas were included. These derived from 138 books written from 206 CE to 1948. These formulas included 5,018 ingredients (mean, 9 ingredients/formula) comprising 243 different natural products. When these text-mining results were compared to the 18 formulas recommended in a modern Chinese Medicine clinical practice guideline, 44% of the herbal formulas were the same. This suggests considerable continuity in the clinical application of these herbs between classical and modern Chinese medicine practice. Of the 15 herbs most frequently used as ingredients of the classical formulas, all have received research attention, and all have been reported to have anti-inflammatory effects. Two of these 15 herbs have already been developed into new anti-RA therapeutics—sinomenine from *Sinomenium acutum* (Thunb.) Rehd. & Wils and total glucosides of peony from *Paeonia lactiflora* Pall. Nevertheless, there remains considerable scope for further research. This text-mining approach was effective in identifying multiple natural product candidates for future research.

## 1. Introduction

Rheumatoid arthritis (RA) is a chronic inflammatory autoimmune disease characterized by multijoint swelling and pain and destruction of the synovial joints, leading to severe disability and increased mortality [1–3]. The global prevalence was estimated at 0.24% but it is higher in some populations [4], with 0.5–1% of adults in the United States being affected [5]. Over the last decade, the optimal use of disease modifying antirheumatic drugs (DMARDs) [6, 7] and the increasing availability of new biological agents [8, 9] have enhanced the success of RA management. Traditional treatment methods are widely used in China often in combination with DMARDs and/or biologics [10]. This is likely due to a combination of concerns about the side effects of DMARD combination therapy, the high cost of biological agents in China, the ready availability of traditional treatments in the hospital system [11], and public awareness of the increasing literature on the evidence base for traditional treatments such as herbal formulations and acupuncture [12–16].

Clinical guidelines for prescribing traditional medicines for RA provide criteria for differentiating the Chinese medicine syndromes and selecting appropriate multi-ingredient formulations which are typically administered in the form of decoctions, granules, capsules, and pills [17]. In addition, manufactured medicines based on extracts of plants used traditionally for joint pain have been developed and evaluated in clinical trials [18, 19].

Along with the increasing application of clinical trial methodologies for the evaluation of traditional medicines for RA and other diseases, there has been increasing attention to the systematic assessment of the premodern and classical medical literature using text-mining approaches [20]. Such studies have focused on drug discovery from compounds found in the natural products used in traditional medicines [21–23]; identification of instances of long-term traditional use of natural products for certain diseases or symptoms [24–27]; the logic underlying ancient acupuncture prescriptions [28]; and investigations of continuities and differences between the classical and modern Chinese medicine approaches to certain diseases [29, 30]. It has been proposed that long-term traditional use could be considered as a source of evidence [31], and a “whole-evidence” approach to evidence-based Chinese medicine could concludes systematic searching of the classical literature as one component [32].

This text-mining study identifies traditional formulations and their constituent natural products that have been used for conditions consistent with RA during the classical and premodern period (until 1949), compares these with the approaches recommended in contemporary guidelines for the application of herbal formulations in RA management, and examines the contemporary research into the natural products most frequently used in the traditional formulations. The study aimed to identify prospects for future clinical and experimental studies, which may lead to the development of new treatments for rheumatoid arthritis.

## 2. Materials and Methods

We searched the Encyclopaedia of Traditional Chinese Medicine (*Zhong Hua Yi Dian*, 5th edition) an electronic database which contains the full texts of over 1150 medical books spanning more than 2000 years [33]. This source was selected because it was the most comprehensive collection available in electronic format [34, 35].

The procedures for text-mining have been detailed elsewhere [36]. There is no single term in the premodern and classical Chinese literature that directly corresponds to the modern conception of RA; however, descriptions of the clinical features of RA have been included under certain traditional terms. Therefore, multiple search terms were selected based on medical nomenclatures [37, 38], clinical practice guidelines [17, 39], textbooks [40, 41], and specialist books [42–44]. Preliminary searches were conducted to determine terms that located passages of text that were suggestive of RA. After discarding unproductive terms, the following Chinese terms for classical disease names and symptoms were used to search the literature: *bi* “arthritis” or “painful blockage”, *li jie* “joint disease”, *tong feng* “painful wind”, *he xi feng* “crane's knee wind”, *bai hu bing* “white tiger disease”, *ji zhua feng* “chicken's claw wind”, *gu chui feng* “drum stick wind,” and *wang* “lameness”. Each term was searched in the *Zhong Hua Yi Dian* (ZHYD) database, and all passages of text identified by these terms were copied to Microsoft Excel spreadsheets (by X.X, B.H.M), together with the identity of the source book and all relevant information on the disorder and intervention. A passage of text that included one or more of the search terms together with an herbal intervention for the disorder was considered a single citation. Duplications were identified and removed. Inclusion and exclusion criteria were used to identify conditions whose signs and symptoms were consistent with the features of rheumatoid arthritis. Each passage was read and allocated codes (by XX, B.H.M).

The inclusion criteria were (1) a specific herbal intervention for oral administration comprising one or more ingredients intended as a treatment for one or more of the search terms and (2) the primary condition had symptoms of joint pain and/or joint swelling, and/or limited joint function. Citations were excluded if the condition (1) had sudden or recent onset (trauma, fever, epidemic or seasonal disorder); (2) was specific to children, teenagers or females; (3) was likely due to a cerebrovascular accident (e.g., *zhong feng*, stroke, paralysis); or (4) was likely due to other rheumatoid disease (e.g., gout, osteoarthritis).

## 3. Results and Discussion

**Figure 1:**
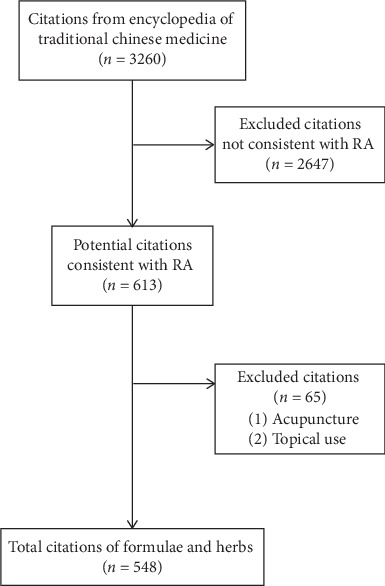
Flowchart of the search and selection process for citations of interventions for disorders consistent with rheumatoid arthritis (RA).

After removal of duplications, 3,174 citations were considered, and 548 citations of traditional formulas were included (Figure 1). The most commonly used search term was *bi* (258 citations) followed *li jie* (175 citations), *tong feng* (89 citations), and *he xi feng* (*n* = 14) but all terms were productive of citations that could have referred to RA. (TABLE SUPP 1). The citations were derived from 138 different books written from circa (ca.) 206 CE to 1948. Most of the books were written during the Ming (1369–1644) and Qing (1645–1911) dynasties (Table 1). *Prescriptions for Universal Relief* (*Pu Ji Fang* c.1406), which is the largest book in ZHYD, provided 56 citations. The next productive book was the *Compendium of Medicine* (*Yi Xue Gang Mu* c. 1565) with 28 citations.

### 3.1. Example Citations

The following three citations have been translated as examples. *Prescriptions for Universal Relief* (volume 120) said that the formula *Niu bang zi san* “treats *li jie* caused by wind and hotness, with pain and swelling of the fingers, back and shoulders, and/or both knees.” In *Compendium of Medicine* (volume 12) in the section on *bi* syndrome, it is recorded that the formula *He xue san tong tang* was used for “someone with pain and swelling in all 10 fingers that appeared one by one, and also in the knees (left then right), with attacks that can last three to five days which are alleviated in the daytime.” The book *Lei Zheng Zhi Cai* (ca.1839) mentioned the use of the formula *Wu tou tang* as a treatment for “*li jie feng* with pain in joints all over the body, just like being bitten by tiger, which is why it is also called white tiger *li jie* (*bai hu lie jie*). The symptoms are contracture and swelling of fingers, severe pain, and even limitation of function.”

### 3.2. Frequencies of Formulas and Their Constituent Ingredients

The citations referred to 98 unnamed herbal formulas and 137 different formula names. *Wu tou tang* (*n* = 35) was the most common formula name, followed by *Gan cao fu zi tang* (*n* = 28). These were early formulae, deriving from the book *Jin Gui Yao Lue Fang Lun* (ca. 206). The next most frequent were *Gui zhi shao yao zhi mu tang* (*n* = 24), and *Si wu tang* including modified versions (*n* = 21) (Table 2).

All the formulas included 5,018 ingredients (mean, 9 ingredients/formula) comprising 243 different natural products. The most frequently used were root of *Glycyrrhiza uralensis* Fisch (*gan cao*, *n* = 286), rhizome of *Zingiber officinale* Rosc (*jiang*, *n* = 209), root of *Angelica sinensis* Oliv. Diels (*dang gui*, *n* = 183), root of *Paeonia lactiflora* Pall. (*shao yao*, *n* = 182), root of *Saposhnikovia divaricata* Turcz. Schischk (*fang feng*, *n* = 165), bark of *Cinnamomum cassia* Presl (*gui zhi*, *n* = 155), aerial parts of *Ephedra sinica* Stapf (*ma huang*, *n* = 151), rhizome of *Atractylodes macrocephala* Koidz (*bai zhu*, *n* = 147), root of *Ligusticum chuanxiong* Hort. (*chuanxiong*,133), and *Notopterygium incisum* Ting ex H. T. Chang (*qiang huo*, *n* = 128) (Table 3). From the Chinese medicine perspective, herbs are traditionally classified by flavour (*wei*), nature (*xing*), and channel tropism (*gui jing*) [45]. Herbs can have multiple flavours and tropisms but one nature. The flavours of the most frequent herbs were bitter (*n* = 18), pungent (*n* = 17) and/or sweet (*n* = 13). More were warm (*n* = 14) than cold (*n* = 7) in nature. The main channel tropisms were the Spleen (*n* = 18), Liver (*n* = 15), Lung (*n* = 13), Heart (*n* = 12), and Kidney (*n* = 10) channels (TABLE SUPP 3).

### 3.3. Comparison with Contemporary Clinical Practice Guidelines

In the recent clinical practice guideline for RA [17] which is currently used by Chinese medicine doctors, 18 different herbal formulas were recommended based on the differentiation of RA into eight Chinese medicine “syndromes” or “patterns” [46]. Although a syndrome differentiation approach was not specified in the citations from the classical books, in some cases, we can infer the likely syndrome from the causative factors and/or the symptoms and signs mentioned in the citations. Of the formulas in the guidelines, eight were included in the ZHYD results: *Qiang huo sheng shi tang*, *Wu tou tang*, *Gui zhi shao yao zhi mu tang*, *Dang gui nian tong tang*, *Er miao san*, *Huang qi gui zhi wu wu tang*, *Du huo ji sheng tang*, and *San bi tang*. These related to five of the eight syndromes. Six additional formulas related to those in the guidelines were also identified (Table 4).

Although some of the more frequent classical formulae in Table 2 were absent in Table 4, the ingredients of these two lists of formulae showed considerable overlap. Of the ingredients of the formulae in the 2018 guidelines (Table 4), all except two also appeared as an ingredient in at least one of the classical formulas (allowing for differences in names). This indicates that while the formula names varied considerably, the ingredients of the modern and classical formulas tended to be drawn from a similar pool of natural products (mostly plants).

### 3.4. Discussion of Main Results

This text-mining study identified citations from the full texts of ancient and premodern Chinese medical books included in the ZHYD that provided orally administered interventions for conditions with the clinical symptoms of joint pain and signs and symptoms that were suggestive of RA. The two most frequently cited formulas were from the earliest included book, and both these formulas are included in contemporary clinical practice guidelines. Almost 50% (44%, 8) of the 18 formulas in contemporary guidelines (including modified versions) were the same as formulas found in the citations from classical and premodern books, indicating considerable continuity in Chinese medicine practice for joint pain and dysfunction that were broadly consistent with the clinical symptoms of RA. However, we cannot retrospectively diagnose cases from the historical literature with any certainty so, despite our selection criteria, some of these citations may have referred to other forms of arthritis and/or joint pain due to other pathophysiology. It is important to note that the formulas and herbs were not specific to RA and could be used for other forms of arthritis, so it is a reasonable conclusion that RA was likely to have been within the scope of usage of the included herbs and formulas.

Of the 28 individual herbs that appeared frequently in the formulas from the classical and premodern literature, a little more than half (16) are listed in the contemporary Chinese pharmacopeia [47] with arthritic conditions as a primary indication. In addition, close to 100% of the ingredients of the 18 formulas in the clinical guideline were also used as ingredients in the classical formulas. This indicates there has been considerable continuity in the use of these herbs until modern times.

From the perspective of Chinese medicine, the traditional disorder “*bi*” was mainly due to the pathogens Wind (*feng*), Cold (*han*), and Dampness (*shi*) causing blockages, although some types are characterized as Dampness-heat (*shi re*) [48, 49]. Herbs classified as bitter (*ku*) can dry Dampness, with bitter-warm herbs being used for Cold-dampness types of the disorder and bitter-cold herbs being used for Dampness-heat types [50]. There were slightly more bitter-warm/hot herbs (*n* = 9) than bitter-cold herbs (*n* = 7). Pungent herbs are used to disperse pathogens such as Wind and/or move blockages to relieve pain [50]. Most were pungent-warm (*n* = 15), which is typical of herbs in this category. Sweet herbs are mainly used for debility and chronic conditions. In addition, they are often combined with other herbs to “harmonise” (*he*) their effects and assist in relieving pain [50]. In terms of the tropism, the Spleen channel has associations relevant to this disorder including Dampness which shows as swelling in this condition and lack of nourishment to the muscles of the four limbs which is a feature of chronic conditions. The Liver channel is associated with disorders of the connective tissue, lack of nourishment of the tendons and joints, and loss of normal flow of *qi* and blood leading to pain. The other channel traditionally associated with this disorder is Kidney which is associated with the condition of the bones [48, 49]. These traditional characteristics of the herbs found in the classical and premodern literature tended to reflect the viewpoint of modern textbooks. In addition, they suggest that the types of arthritis included Cold-damp and Damp-heat syndromes, although Cold-damp syndromes may have predominated. The top two channel tropisms were in accord with modern textbooks, but it is interesting to note that the Lung and Heart channels were also frequent. These are not usually associated with arthritis in traditional books. However, from a modern perspective, disorders of the pulmonary and cardiac systems are often comorbid with RA [5], and traditional formulations tend to combine ingredients to address both articular and extra-articular symptoms.

In this study, we have used frequency of appearance of a formula as a method of listing. However, frequency should not be misconstrued as an indication of effectiveness. In the case of formula ingredients, some were added to manage joint pain and swelling, while others had functional roles including assisting the main herbs to enhance their effects or reduce the adverse effects of some herbs, as guided by Chinese medicine theory. Therefore, the frequency of ingredients was influenced by the individualized clinical practice approach in Chinese medicine, and it does not indicate clinical effectiveness in the evidence-based healthcare context.

### 3.5. Modern Research into Herbs Used Frequently in the Classical Literature

Some of the classical formulas (Table 4) have received research attention in clinical trials for RA. These include *Wu tou tang* [51], *Wu ji san* [52], *Gui zhi shao yao zhi mu tang* [53], *Huang qi gui zhi wu wu tang* [54], *Dang gui nian tong tang* [55], and *Du huo ji sheng tang* [56]. Some formulations have been investigated in experimental studies including *Wu tou tang* [57, 58] and *Fang ji huang qi tang* [59, 60].

From the perspective of drug discovery and development, herbs and their constituent compounds have long been sources of new molecules and structures [61–63]. To provide a brief overview of research in English literature into the single herbal ingredients, we selected the 15 herbs most frequently included in the classical formulas (see Table 3) and summarized *in-vitro*, *in-vivo,* and human studies with a focus on the reported actions of the herbs and/or their constituent compounds in models relevant to RA (Table 5). The table has been organized according to the traditional herbal names, since this was how the list of herbs was identified, but we have grouped together items that have been processed differently, for example unprocessed licorice root (*sheng gan cao*) and honey-fried licorice root (*zhi gan cao*), and different plant parts when they have similar constituents, for example cinnamon twigs (*gui zhi*) and stem bark (*rou gui*). In some cases, the same traditional name may apply to multiple species from the same genus; for example, the various *Ephedra* species are called *ma huang*. In other cases, the same traditional name could refer to plants from different genera. For example, *niu xi* is mainly sourced from *Achyranthes* species, but *Cyathula officinalis* Kuan is another source, which is now called *chuan niu xi*. Similarly, *fang ji* can derive from the stems of different vines including *Stephania tetrandra* S. Moore and *Sinomenium acutum* (Thunb.) Rehd. & Wils (also called *qing feng teng*), but unfortunately it has also been sourced from toxic *Aristolochia* species leading to many cases of poisoning [64]. In Table 5, we have listed the main traditional names and source species for the herbs, but this is not an exhaustive list since multiple traditional names exist, the species used may have changed over time, and there can be regional variation in the preferred species. These issues all present challenges for drug discovery when traditional Chinese literature is used. However, many issues can be resolved by consulting traditional pharmacopeia from different periods, especially those with good-quality illustrations such as *Shi Zheng Lei Da Guan Ben Cao* (ca. 1108), good editions of *Ben Cao Pin Hui Jing Yao* (ca. 1505) and *Ben Cao Gang Mu* (ca. 1593), together with modern comprehensive works such as *Zhong Hua Ben Cao* [65].

With regard to research into the herbs in Table 5, two plants have already been developed into therapies for RA. Sinomenine is derived from *Sinomenium acutum* (Thunb.) Rehd. & Wils [139—140] and total glucosides of peony are from *Paeonia lactiflora* Pall., [141–143]. Interestingly, the plant *Tripterygium wilfordii* Hook F (*lei gong teng*) which has also been developed into therapies for RA [144–147], did not appear in any of the formulas located in this text-mining study.

Anti-inflammatory effects have been reported for each of the 15 herbs. Analgesic and antinociceptive effects have been reported for certain plants, notably the *Aconitum* species, *Paeonia lactiflora* Pall., *Saposhnikovia divaricata* Turcz. Schischk, *Notopterygium incisum* Ting, *Achyranthes bidentata* Bl, and *Sinomenium acutum* (Thunb.) Rehd. & Wils. In reflection of the frequent clinical application of these herbs in combination with conventional medications, some studies have examined combined effects. For example, compounds from *Glycyrrhiza uralensis* Fisch have been reported to enhance the therapeutic effects of NSAIDs and DMARDs [68], and the compound ligustrazine derived from *Ligusticum chuanxiong* Hort. was reported to reduce bone cortex erosion when combined with leflunomide in a clinical study [99]. Besides the discovery of new compounds from these herbs [148], research into novel applications of multiple compounds may provide a further avenue for developing new therapeutics [149].

### 3.6. Limitations and Strengths of the Study

This text-mining study used ZHYD 5^th^ edition as the source for data– to our best knowledge the most comprehensive electronic database of classical literature in Chinese medicine. While this is a large and representative sample of premodern and classical books on Chinese medicine, it does not include every book, so there are some inevitable omissions. In addition, the terms used for searching were limited to eight, so passages of text that did not include any of these terms could not be located. It is likely that some relevant citations would not use these terms since there was no established traditional term specific to the modern diagnostic criteria of RA, so citations that simply described the clinical symptoms of swollen and painful joints would have been missed. However, the search term *bi* has had widespread, long-term use for painful conditions, especially those of the joints, so it is likely that it would have captured most descriptions of disorders consistent with RA. Therefore, the main sources of type 2 errors (false exclusions) relate to the comprehensiveness of the sample and the limited set of search terms leading us to miss some citations of potential relevance to RA.

On the other hand, the resultant data set was large, with 3,174 citations that required assessment (TABLE SUPP 1). Amongst these were citations unlikely to have referred to RA that were captured by the exclusion criteria. Conversely some citations mentioned typical symptoms such as multiple, painful, swollen, and finger joints. However, our modern conception of RA was not shared by doctors in premodern times, so they did not always provide the details we would like. In many citations, there was joint pain but not enough additional detail to distinguish the likely cause. Hence, these were considered as “possible” RA. This is the major source of type 1 errors (false inclusions) in this analysis, since it is likely that such citations could have referred to osteoarthritis or other chronic joint disorders. In the case of the most productive search term, *bi*, only 258 out of 2,147 citations (12%) were considered “possible RA”. This reflects the broad scope of meaning of this traditional term and our requirement that joint pain be specified. In contrast, the term *li jie* yielded a 40% inclusion rate since it was more specific to painful joints. In the case of the term *he xi feng* only 9.3% of citations were included since this condition mainly presents with swelling of the knee consistent with osteoarthritis of the knee. Therefore, our procedures eliminated a considerable proportion of the original data set as not consistent with RA or too unclear for inclusion.

Applying more stringent criteria overall would have further reduced the total number of included citations, at the expense of increasing the number of type 2 errors. We did not take this approach since were interested in identifying candidate formulas for clinical studies and herbs for drug discovery. We were also aware that the same herbal formulas could be used for a range of arthritic disorders, so we did not want to exclude formulas that would have been used for RA plus other disorders. As the results of the experimental studies demonstrate, this approach was productive in identifying herbs that have received varying degrees of research attention of relevance to RA, since all the herbs in Table 5 have histories of use for painful joint disorders and have shown biological activities relevant to RA management in recent studies. Moreover, the identified herbs have all been used in humans, so any toxicity issues are likely to have been identified. Nevertheless, the pathway from identifying candidates to the development of new interventions still requires extensive preclinical and clinical research [150].

## 4. Conclusions

This text-mining study of a large sample of the premodern and classical Chinese medicine literature identified herbal interventions for joint disorders associated with possible RA. When the results for the formulas were compared with the recommendations in the current clinical practice guidelines for the treatment of RA using Chinese herbal medicine, there was considerable overlap, suggesting considerable continuity between historical and modern clinical practice. At the level of the individual ingredient, all the top 15 herbs frequently used in the classical formulas have also been reported to demonstrate anti-inflammatory activity in experimental studies and some have already received considerable research attention as candidates for RA drug discovery. These results suggest this text-mining approach was productive in identifying potential natural products for further research.

## Figures and Tables

**Table 1:** Citations of interventions for conditions broadly consistent with rheumatoid arthritis by historical period.

Historical period (dynasty)^1^	Frequency (percentage)
Before Tang dynasty (−617)	1 (0.20)
Tang and 5 dynasties (618–959)	4 (0.70)
Song Jin dynasties (960–1271)	52 (9.5)
Yuan dynasty (1272–1368)	11 (2.0)
Ming dynasty (1369–1644)	245 (44.7)
Qing dynasty (1645–1911)	221 (40.3)
Ming Guo period (1912–1949)	14 (2.6)
Total	548 (100)

^1^The dividing points between dynasties are open to interpretation, so the years have been adjusted to avoid overlap. When authors lived across two dynasties, the dynasty usually cited for the book was adopted. See May et al. [36] for how book years were determined.

**Table 2:** Herbal formulas frequently identified in citations of treatments of conditions broadly consistent with rheumatoid arthritis.

Formula name^1^	Frequency(*n*)^2^	Formula ingredients^3^; first book in group of citations^4^ (year)^5^
Wu tou tang	35	Ma huang, Shao yao, Huang qi, Gan cao, Chuan wu; *Jin Gui Yao Lue Fang Lun* (ca. 206)
Gan cao fu zi tang	28	Gan cao, Fu zi, Bai zhu, Gui zhi; *Jin Gui Yao Lue Fang Lun* (ca. 206)
Gui zhi shao yao zhi mu tang	21	Gui zhi, Shao yao, Gan cao, Ma huang, Sheng jiang, Bai zhu, Zhi mu, Fang feng, Fu zi; *Zheng Yin Mai Zhi* (ca. 1641)
Si wu tang (modified)	16 (21)	Cang zhu, Huang bai, Fu zi, Gan cao, Ma huang, Tao ren, Sheng Jiang, Niu xi, Chen pi, Gan cao, Bai zhi, Huang qin, Long dan cao; *Ge Zhi Yu Lun* (ca. 1347)
Fang ji huang qi tang	12 (13)	Fang ji, Huang qi, Bai zhu, Gan cao, Sheng jiang, Da zao; *Mi Zhuan Zheng Zhi Yao Jue Ji Lei Fang* (ca. 1443)
Wu ji san	8 (12)	Cang zhu, Jie geng, Zhi ke, Chen pi, Shao yao, Bai zhi, Chuanxiong, Dang gui, Gan cao, Rou gui, Fu ling, Hou pu, Gan jiang, Ma huang, Quan xie, Ma huang, Sheng jiang; *Pu Ji Fang* (ca. 1406)
Fu zi ba wu tang	11	Fu zi, Gan jiang, Shao yao, Bai zhu, Fu ling, Gui xin, Ren shen, Gan cao; *Yi Fang Xuan Yao* (ca. 1495)
Niu bang zi san	10	Niu bang zi, Dan dou chi, Qiang huo, Sheng di huang, Huang qi; *Pu Ji Fang* (ca. 1406)

^1^Formulas with the same name can vary in their ingredients, and the same combination of ingredients may have different names. In these data, formulas with the same core ingredients and the same name are grouped together, while those with different main ingredients are separated. Also, formulas with the same ingredients but different names have been grouped together. ^2^The frequency is for the name in the left column, and the number in parentheses includes modified versions of the formula. ^3^Formulae that include an endangered species as a primary ingredient have been excluded, when the ingredient was minor and substitutable—the substitute has been recorded, when minor but not substitutable the ingredient has been excluded. For scientific names of ingredients written in Pin Yin and Chinese characters for traditional medicines and book names, see glossary in supplementary file. ^4^First book in group of citations; i.e., the oldest book within the group of included citations, not the first book that included the formula. ^5^Dates are approximate.

**Table 3:** Natural products frequently used in formulas for conditions broadly consistent with rheumatoid arthritis.

Scientific name (part)^1^	Chinese traditional name	Frequency (n)	In contemporary pharmacopoeia^2^
*Glycyrrhiza uralensis* Fisch. (root)	Gan cao	286 (3 sheng gan cao, 26 zhi gan cao)	No
*Zingiber officinale* Rosc. (rhizome)	Jiang	209 (115 sheng jiang, 28 gan jiang, 6 pao jiang)	No
*Angelica sinensis* Oliv. Diels (root)	Dang gui	183	Yes
*Paeonia lactiflora* Pall. (root)	Shao yao	182 (28 chi shao yao, 43 bai shao yao)	No
*Saposhnikovia divaricata* Turcz. Schischk (root)	Fang feng	165	Yes
*Cinnamomum cassia* Presl. (bark)	Gui zhi	155	Yes
*Ephedra sinica* Stapf. (herb)	Ma huang	151	No
*Atractylodes macrocephala* Koidz. (rhizome)	Bai zhu	147	No
*Ligusticum chuanxiong* Hort. (root)	Chuan xiong	133 (1 xiong qiong, 2 tai qiong)	Yes
*Notopterygium incisum* Ting (root and rhizome)	Qiang huo	128	Yes
*Poria cocos* Schw. Wolf (sclerotium)	Fu ling	127 (23 chi fu ling, 13 bai fu ling, 7 fu shen)	No
*Achyranthes bidentata* Bl; *Cyathula officinalis* Kuan (root)	Niu xi	118 (9 huai niu xi, 9 chuan niu xi)	Yes
*Aconitum carmichaelii* Debx; *A*. *kusnezoffii* Reichb. (root)	Wu tou	102 (57 chuan wu, 8 cao wu)	Yes
*Astragalus membranaceus* (Fisch.) Bge. *var*. *mongholicus* (Bge.) Hsiao (root)	Huang qi	92	Yes
*Stephania tetrandra* S. Moore; *Sinomenium acutum* (Thunb.) Rehd. & Wils.^3^ (caulis)	Fang ji	88 (6 han fang ji, 2 mu fang ji)^4^	Yes
*Panax ginseng* C. A. Mey (root)	Ren shen	81	No
*Atractylodes lancea* (Thunb.) DC (rhizome)	Cang zhu	81	Yes
*Angelica dahurica* (Fisch. ex Hoffm.) Benth. et Hook. f (root)	Bai zhi	78	Yes
*Aconitum carmichaelii* Debx (root)	Fu zi	72 (7 sheng fu zi, 1 shu fu zi, 57 pao tian xiong, 3 da fu zi)	Yes
*Angelica pubescens Maxim*. f. biserrata Shan et Yuan (root)	Du huo	69	Yes
*Scutellaria baicalensis* Georgi (root)	Huang qin	65	No
*Citrus reticulata* Blanco (peel)	Chen pi	60	No
*Prunus persica* (L.) Batsch (seed)	Tao ren	59	No
*Rehmannia glutinosa* Libosch. (root)	Di huang	56 (30 sheng di huang, 18 shu di huang, 3 gan di huang)	No
*Cinnamomum cassia* Presl (bark)	Rou gui	55	Yes
*Gentiana macrophylla* Pall (root)	Qin jiao	54	Yes
*Phellodendron amurense* Rupr (bark)	Huang bai	53	No
*Clematis chinensis* Osbeck; *C*. *hexapetala* Pall.; *C*. *manshurica* Rupr.	Wei ling xian	45	Yes

^1^Scientific names are based on *Pharmacopoeia of the People*'*s Republic of China* 2010 and/or *Great Compendium of Chinese Medicines*. ^2^Clinical applications based on *Pharmacopoeia of the People*'*s Republic of China* specified use for arthritic/rheumatic conditions. ^3^Also called *qing feng teng*. ^4^This herb must not be sourced from *Aristolochia* species.

**Table 4:** Formulas for rheumatoid arthritis in the contemporary clinical guideline and corresponding formulas in the search results.

Syndromes in 2018 guideline	Formulas in 2018 guideline^1^	Formulas in ZHYD search^1^
(1) Bi syndrome due to wind and coldness^2^	Qiang Huo Sheng Shi Tang; Juan Bi tang; Da qin jiao tang.	Qiang Huo Sheng Shi Tang (*n* = 5). Related formulas: Da qiang huo tang (*n* = 8); Qin jiao tang (*n* = 2).
(2) Bi syndrome due to cold and dampness	Wu tou tang; Gui zhi shao yao zhi mu tang; Ma huang fu zi xi xin tang.	Wu tou tang (*n* = 35); Gui zhi shao yao zhi mu tang (*n* = 21)
(3) Bi syndrome due to dampness and heat	Xuan bi tang; Dang gui nian tong tang; Er miao san.	Dang gui nian tong tang (*n* = 5); Er miao san/wan (*n* = 7).
(4) Bi syndrome due to phlegm and blood stasis	Shuang he tang.	Related formulas: Si wu tang (*n* = 21); Wu ji san (*n* = 12); Er chen tang (*n* = 5).
(5) Bi syndrome due to blood stasis	Shen tong zhu yu tang; Tao hong yin.	Related formula: He xue san tong tang (*n* = 4).
(6) Bi syndrome due to deficiency of *qi* and blood	Huang qi gui zhi wu wu tang; Shi quan da bu tang; Gui pi tang.	Huang qi gui zhi wu wu tang, also called Huang qi wu wu tang (*n* = 2)
(7) Bi syndrome due to deficiency of liver and kidney	Du huo ji sheng tang; San bi tang.	Du huo ji sheng tang (*n* = 4); San bi tang (*n* = 1).
(8) Bi syndrome due to deficiency of *qi* and *yin*^3^	Si shen jian.	None relevant

^1^See supplementary 2 for the Chinese characters. ^2^Bi syndrome is a broad traditional diagnostic category that includes arthritis, but here it refers to RS. ^3^This syndrome is for RA with Sjogren syndrome, which often has symptoms of dry eye or dry mouth. ZHYD: *Zhong Hua Yi Dian*.

**Table 5:** Reported biological actions relevant to rheumatoid arthritis of the higher frequency herbs.

Traditional name(s) in pin yin	Botanical source(s)	Material tested: extract/compound	Reported actions relevant to RA
Gan cao, Sheng gan cao, Zhi gan cao	*Glycyrrhiza uralensis* Fisch; *G*. *glabra* L. (Licorice)	Liquiritin [66], glycyrol [67], glycyrrhizin, glycyrrhetinic acid [68]	(i) Anti-inflammatory [66–68], (ii) restrained angiogenesis [66], (iii) down-regulated autoimmune reactions [67], (iv) enhanced therapeutic effects of NSAIDs/DMARDs [68]
Jiang, Sheng jiang, Gan jiang	*Zingiber officinale* Rosc (Ginger)	Extracts [69, 70], essential oils [71], shogaols, gingerols [70], 6-gingerol [72]	(i) Anti-inflammatory [69–71], (ii) Inhibited inflammation-associated osteoclast differentiation [72]
Dang gui	*Angelica sinensis* Oliv. Diels	Extract [73], ethyl acetate fraction [74], ferulic acid, Z-ligustilide [75], calycosin [76]	(i) Anti-inflammatory [75], (ii) Inhibited rheumatoid synovial fibroblast proliferation [74], (iii) Potential interleukin 6R inhibitor [76], (iv) Inhibited osteoclast differentiation [73]
Shao yao, Bai shao yao, Chi shao yao	*Paeonia lactiflora* Pall.	Total glucosides [77–80], paeoniflorin [81], paeoniflorin-6′-O-benzene sulfonate [81, 82]	(i) Anti-inflammatory, analgesic [77, 80], (ii) Prevented bone loss/joint destruction [79, 80], (iii) Immune regulatory [78, 81], (iv) Inhibited synovial hypertrophy and neovascularization [80], (v) enhanced effects of methotrexate [82]
Fang feng	*Saposhnikovia divaricata* Turcz. Schischk^1^	Extract [83], chromone extract [84], chromones: divaricatol, ledebouriellol and hamaudol [85], polysaccharide [86]	(i) Anti-inflammatory [83, 84, 86], (ii) Analgesic [85, 87], (iii) Immunomodulatory [87]
Gui zhi, Rou gui	*Cinnamomum cassia* Presl	Extracts [88–90], cinnamic acid [90], cinnamomulactone [91]	(i) Anti-inflammatory [90], (ii) Anti-arthritic, reduces joint swelling [88], (iii) Inhibitory activity against matrix metalloproteinases [91], (iv) Improved clinical symptoms and inflammatory markers [89]
Ma huang	*Ephedra sinica* Stapf, other *Ephedra* spp.	Polysaccharides [92, 93]	(i) Anti-inflammatory, immuno-suppressive [92, 93]
Bai zhu	*Atractylodes macrocephala* Koidz	Extract [94], atractylone [95], atractylenolide I [94], atractylenolide III [96], Sesquiterpenoids [97]; three new compounds [98]	(i) Anti-inflammatory [94–98]
Chuan xiong, xiong qiong	*Ligusticum chuanxiong* Hort.	Ligustrazine [99], Z-ligustilide, senkyunolide A [100], ligustilides [101]	(i) Anti-inflammatory [100, 101], (ii) reduced bone cortex erosion when combined with leflunomide [99]
Qiang huo	*Notopterygium incisum* Ting	Extract [102], volatile compounds [103], polyacetylenes [104], notopterol [105]	(i) Anti-inflammatory [103, 104, 106], (ii) Analgesic [105], (iii) reduced swelling [102], (iv) Anti-angiogenic [103]
Fu ling, Fu shen, Chi fu ling	*Poria cocos* Schw. Wolf	Polysaccharides [107, 108], pachymic acid, dehydrotumulosic acid [109] triterpenoids [110]	(i) Anti-inflammatory [108–110], (ii) Immunomodulatory [107, 109]
Niu xi, Huai niu xi, Chuan niu xi	(1). *Achyranthes bidentata* Bl; *A*. *aspera* L.; (2). *Cyathula officinalis* Kuan	(1). Extracts [111, 112], polysaccharide [113]; (2). Extract [114]	(1). anti-inflammatory [111, 112], (i) Analgesic [111], (ii) Suppressed osteoclastogenesis and bone resorption [113]; (2). down-regulated matrix metalloproteinase-13, (i) Chondroprotective [114]
Wu tou, Chuan wu, Cao wu, Fu zi	*Aconitum carmichaelii* Debx; *A*. *kusnezoffii* Reichb.^3^	Extracts [115–118], alkaloids [119], benzoylaconitine [120]	(i) Anti-inflammatory [115, 117, 120], (ii) Analgesic [116, 118, 119]
Huang qi	*Astragalus membranaceus* (Fisch.) Bge. *var*. *mongholicus* (Bge.) Hsiao	Extracts [121, 122], astragalosides [123], astragaloside IV [124], astragalus glycoprotein [125], polysaccharides [126, 127]	(i) Anti-inflammatory [121–124, 126, 127], (ii) Analgesic [122], (iii) enhanced autophagy [126], (iv) enhanced synovial apoptosis [127], (v) Improved pathological state of synovial membranes [125]
Fang ji, Han fang ji, mu fang ji	(1). *Stephania tetrandra* S. Moore; (2). *Sinomenium acutum* (Thunb.) Rehd. & Wils.^4^	(1). Extract [128, 129], tetrandrine [130–132]; (2). Sinomenine [133–138]	(1). -anti-inflammatory [128, 131], (i) Antifibrosis [128], (ii) Immuno-suppressive [130], (iii) Suppressed excessive granulocyte activation [129], (iv) Inhibited migration and invasion of fibroblast-like synoviocytes [132], (v) Ameliorated arthritis symptoms [131]; (2). - anti-inflammatory [133, 134, 138], (i) reduced arthritis symptoms [137], (ii) reduced cartilage damage [133, 134], (iii) reduced bone erosion [134], (v) Inhibited angiogenesis [134], (vi) Antinociceptive [135, 136]

^1^Also called: *Ledebouriella divaricata* (Turcz.) Hiroe. ^2^Also called *Ligusticum wallichii* Franch. ^3^These species contain the toxic alkaloid aconitine. ^4^Also called: *Cocculus orbiculatus* (L.) DC. and *qing feng teng*.

## Data Availability

The database used in this study is commercially available. The datasets used for this study are available from the authors upon reasonable request.

## Supplementary Materials

Supplementary Materials TABLE SUPP 1: frequency of citations for formula interventions identified by the search terms at different stages in the selection procedure. SUPP 2: glossary of Pin yin, Chinese, and scientific names for traditional medicines, books, formulas, and other terms cited in the text and tables. TABLE SUPP 3: traditional properties of the most frequent herbs: channel tropism, flavour, nature.
