# Supplementary material for: Chinese Herbal Medicines for Rheumatoid Arthritis: Text-Mining the Classical Literature for Potentially Effective Natural Products
Source: Evid Based Complement Alternat Med. 2020 Apr 28;2020:7531967. doi: 10.1155/2020/7531967 (PMC7206865; doi:10.1155/2020/7531967)
Supplement: Supplementary Materials — TABLE SUPP 1: frequency of citations for formula interventions identified by the search terms at different stages in the selection procedure. SUPP 2: glossary of Pin yin, Chinese, and scientific names for traditional medicines, books, formulas, and other terms cited in the text and tables. TABLE SUPP 3: traditional properties of the most frequent herbs: channel tropism, flavour, nature. [file 7531967.f1.pdf]

Chinese herbal medicines for Rheumatoid Arthritis: text-mining the Classical literature for potentially effective natural products

Xuan Xia[1,2], Brian H May[3], Anthony Lin Zhang[3], Xinfeng Guo[1,2], Chuanjian Lu[1,2], Charlie C. Xue[1,3], Qingchun Huang[1,2]

Affiliation

1. The Second Clinical College of Guangzhou University of Chinese Medicine, Guangzhou, China

2. Department of Rheumatology and Immunology, Guangdong Provincial Hospital of Chinese Medicine, Guangzhou, China.

3. China-Australia International Research Centre for Chinese Medicine, RMIT University, Bundoora, Victoria, Australia.

Supplementary materials

TABLE SUPP 1: Frequency of citations for formula interventions identified by the search terms at different stages in the selection procedure

**Search term in pin yin**	**Search term in Chinese**	**All data** (after duplicate removal)	**Included citations**	**Earliest dynasty**	**Earliest Book**
1: Bi	痹	2147	258	Dong han dynasty	*Jin Gui Yao Lue*
2: Li jie	历节	473	175	Song dynasty	*Tai Ping Sheng Hui Fang*
3: Tong feng	痛风	363	89	Song dynasty	*Ren Zhai Zhi Zhi Fang*
4: He xi feng	鹤膝风	150	14	Song dynasty	*Sheng Ji Zong Lu*
5: Bai hu bing	白虎病	11	5	Ming dynasty	*Yi Xue Gang mu*
6: Ji zhua feng	鸡爪风	11	2	Yuan dynasty	*Ren Shu Bian Lan*
7: Gu chui feng	鼓槌风	9	3	Ming dynasty	*Sun Wen Heng Yi An*
8: Wang	尪	10	2	Qing dynasty	*Yi Zong Jin Jian*
Total		3174	548		

SUPP 2: Glossary of Pin yin, Chinese and scientific names for traditional medicines, books, formulas and other terms cited in the text and tables

Ben Cao Pin Hui Jing Yao 本草品汇精要 (c. 1505), book name

Ben Cao Gang Mu 本草纲目 (c. 1593), book name

Bai fu ling 白茯苓, *Poria cocos* (Schw.) Wolf [sclerotium] (see Table 3)

bai hu bing 白虎病, disease name 'white tiger disease'

bai hu li jie 白虎历节, disease name 'white tiger joint disease'

Bai shao yao 白芍药 P*aeonia lactiflora* Pall. [radix] (see Table 3)

Bai zhu 白术, *Atractylodes macrocephala* Koidz [rhizome] (see Table 3)

Bai zhi 白芷, *Angelica dahurica* Fisch. ex Hoffm.Benth.et Hook.f [root] (see Table 3)

bi 痹, disease name 'arthritis' 'painful obstruction'

Cao wu 草乌, *Aconitum kusnezoffii* Reichb. [root] (see Table 3)

Chuan niu xi 川牛膝, *Cyathula officinalis* Kuan [root] (see Table 3)

Chuan wu 川乌, *Aconitum carmichaelii* Debx [mother root]

Cang zhu 苍术, *Atractylodes lancea* Thunb.DC [rhizome] (see Table 3)

Chen pi 陈皮, *Citrus reticulata* Blanco [skin, peel] (see Table 3)

Chi fu ling 赤茯苓, *Poria cocos* (Schw.) Wolf [sclerotium] (see Table 3)

Chi shao yao 赤芍药 *Paeonia lactiflora* Pall. [radix] (see Table 3)

Chuan xiong 川芎, *Ligusticum wallichii* Franch [root] (see Table 3)

Dang gui 当归, *Angelica sinensis* Oliv. Diels [root] (see Table 3)

Dan dou chi 淡豆豉, *Glycine max* L. Merr [seed]

Da qiang huo tang 大羌活汤: formula name (see Table 2)

Da qin jiao tang 大秦艽汤: formula name (see Table 4)

Dang gui nian tong tang 当归拈痛汤: formula name (see Table 4)

Da zao 大枣, *Ziziphus jujuba* Mill [fruit]

Di huang 地黄, *Rehmannia glutinosa* Libosch. [root] (see Table 3)

Du huo ji sheng tang 独活寄生汤: formula name (see Table 4)

Du huo 独活, *Angelica pubescens Maxim*. f. *biserrat*a Shan et Yuan [root] (see Table 3)

Da fu zi 大附子, *Aconitum carmichaelii* Debx. [root] (see Table 3)

Er miao san 二妙散: formula name (see Table 4)

Er chen tang 二陈汤: formula name (see Table 4)

Fu zi 附子, *Aconitum carmichaelii* Debx [processed root] (see Table 3)

Fang feng 防风, *Saposhnikovia divaricata* Turcz. Schischk [root] (see Table 3)

Fang ji huang qi tang 防己黄芪汤: formula name (see Table 2)

Fang ji 防己, *Stephania tetrandra* S. Moore [caulis] (see Table 3)

Fu shen 茯神, *Poria cocos* (Schw.) Wolf, [sclerotium] (see Table 3)

Fu ling 茯苓, *Poria cocos Schw.*Wolf [sclerotium] (see Table 3)

Fu zi ba wu tang 附子八物汤: formula name (see Table 2)

Gui pi tang 归脾汤: formula name (see Table 4)

Gan cao 甘草, *Glycyrrhiza uralensis* Fisch [root] (see Table 3)

Gan cao fu zi tang 甘草附子汤: formula name (see Table 2)

Gan di huang 干地黄, *Rehmannia glutinosa* Libosch. [root] (see Table 3)

gu chui feng 鼓槌风, disease name 'drum stick wind'

Gui zhi 桂枝, *Cinnamomum cassia* Presl [twig] (see Table 3)

Gui xin 桂心, *Cinnamomum cassia* Presl [bark]

Gui zhi shao yao zhi mu tang 桂枝芍药知母汤: formula name (see Table 2)

Gan jiang 干姜, *Zingiber officinale* Rosc [rhizome]

Ge Zhi Yu Lun 格致余论 (c.1347) book name

he xi feng 鹤膝风, disease name 'crane knee wind'

He xue san tong tang 和血散痛汤: formula name

Han fang ji 汉防己, *Stephania tetrandra* S. Moore [caulis] (see Table 3)

Huai niu xi 怀牛膝, *Achyranthes bidentata* Bl [root] (see Table 3)

Huang qi gui zhi wu wu tang 黄芪桂枝五物汤: formula name (see Table 4)

Hou pu 厚朴, *Magnolia officinalis* Rehd. et Wils; *M. officinalis* Rehd. et Wils. var. *biloba* Rehd. et Wils [Cortex]

Huang qi 黄芪, *Astragalus membranaceus.* Fisch*. Bge. var. mongholicus* Bge.Hsiao [root] (see Table 3)

Huang bai 黄柏, *Phellodendron chinense* Schneid [cortex] (see Table 3)

Huang qin 黄芩, *Scutellaria baicalensis* Georgi [root] (see Table 3)

Jiang 姜, *Zingiber officinale* Rosc. [rhizome] (see Table 3)

Jin Gui Yao Lue Fang Lun 金匮要略方论 (c. 206) book name

Jie geng 桔梗, *Platycodon grandiflorum* Jacq. A. DC [root]

ji zhua feng 鸡爪风, disease name 'chicken's claw wind'

Juan bi tang 蠲痹汤: formula name (see Table 4)

Jing Shi Zheng Lei Da Guan Ben Cao 经史证类大观本草 (c. 1108) book name

Lei feng shi guan jie yan 类风湿关节炎: rheumatoid arthritis

Lei Zheng Zhi Cai 类证治裁 (c.1839) book name

Lei gong teng 雷公藤, *Tripterygium wilfordii* Hook F [all parts]

li jie (bing) 历节(病), disease name 'joint disease'

li jie feng 历节风, disease name 'joint wind', 'articular wind'

Long dan 龙胆, *Gentiana manshurica* Kitage; *G. scabra* Bge; *G. triflora* Pall; *G. rigescens* Franch [rhizome]

Ma huang 麻黄, *Ephedra sinica* Stapf; *E. equisetina* Bge; *E. intermedia* Schrenk et C.A. Mey [aerial parts] (see Table 3)

Mi Zhuan Zheng Zhi Yao Jue Ji Lei Fang 秘传证治要诀及类方 (c.1443) book name

Ma huang fu zi xi xin tang 麻黄附子细辛汤: formula name (see Table 4)

Mu fang ji 木防己, *Cocculus orbiculatus* (L.) DC. [root]

Niu bang zi san 牛蒡子散: formula name (see Table 2)

Niu xi 牛膝, *Achyranthes bidentata* Bl [root] (see Table 3)

Niu bang zi 牛蒡子, *Arctium lappa* L [fructus] (see Table 3)

Pao jiang 炮姜, *Zingiber officinale* Rosc. [processed rhizome] (see Table 3)

Pao tian xiong 炮天雄, *Aconitum carmichaelii* Debx [processed root] (see Table 3)

Pu Ji Fang 普济方 (c.1406) book name: 'Prescriptions for Universal Relief'

Qin jiao tang 秦艽汤: formula name (see Table 4)

Qiang huo sheng shi tang 羌活胜湿汤: formula name (see Table 4)

Quan xie 全蝎, *Buthus martensii* Karsch [scorpio]

Qiang huo 羌活, *Notopterygium incisum* Ting [root and rhizome] (see Table 3)

Qing feng teng 青风藤, *Sinomenium acutum* (Thunb.) Rehd. et Wils [caulis] (see Table 3)

Qin jiao 秦艽, *Gentiana macrophylla* Pall; *G. straminea* Maxim; *G. crassicaulis Duthie* ex Burk; *G. dahurica* Fisch [root] (see Table 3)

Ren shen 人参, *Panax ginseng* C. A. Mey [root] (see Table 3)

Rou gui 肉桂, *Cinnamomum cassia* Presl [cortex] (see Table 3)

San bi tang 三痹汤: formula name (see Table 4)

Sheng di huang 生地黄, *Rehmannia glutinosa* Libosch. [root] (see Table 3)

Sheng fu zi 生附子, *Aconitum carmichaelii* Debx [root] (see Table 3)

Sheng gan cao 生甘草, *Glycyrrhiza uralensis* Fisch [root] (see Table 3)

Shu di huang 熟地黄, *Rehmannia glutinosa* Libosch. [processed root] (see Table 3)

Shu fu zi 熟附子, *Aconitum carmichaelii* Debx [processed root] (see Table 3)

Si shen jian 四神煎: formula name (see Table 4)

Shen tong zhu yu tang 身痛逐瘀汤: formula name (see Table 4)

Shi quan da bu tang 十全大补汤: formula name (see Table 4)

Shi Zheng Lei Da Guan Ben Cao 经史证类大観本草 (ca. 1108) book name

Shao yao 芍药, *Paeonia lactiflora* Pall [root] (see Table 3)

Sheng jiang 生姜, *Zingiber officinale* Rosc [rhizome] (see Table 3)

Si wu tang 四物汤: formula name (see Table 2)

Sheng di huang 生地黄, *Rehmannia glutinosa* Libosch [radix] (see Table 3)

Sheng jiang 生姜, *Zingiber officinale* Rosc. [rhizome] (see Table 3)

Shuang he tang 双合汤: formula name (see Table 4)

Tai qiong 台芎, *Ligusticum wallichii* Franch [root] (see Table 3)

Tao hong yin 桃红饮: formula name (see Table 4)

Tao ren 桃仁, *Prunus persica* L. Batsch [seed] (see Table 3)

tong feng 痛风, disease name 'painful wind'

wang 尪, disease name 'lameness'

Wu tou 乌头, *Aconitum carmichaelii* Debx; *A. kusnezoffii* Reichb. [radix] (see Table 3)

Wu tou tang 乌头汤: formula name (see Table 2)

Wu ji san 五积散: formula name (see Table 2)

Wei ling xian 威灵仙, *Clematis chinensis* Osbeck; *C. hexapetala* Pall; *C. manshurica* Rupr [rhizome] (see Table 3)

Xiong qiong 芎䓖, *Ligusticum wallichii* Franch [root] (see Table 3)

Xuan bi tang 宣痹汤: formula name (see Table 4)

Yi Fang Xuan Yao 医方选要 (c.1495)

Yi Xue Gang Mu 医学纲目 (c. 1565): book name 'Compendium of Medicine'

Zheng Yin Mai Zhi 症因脉治 (c.1641) book name

Zhi gan cao 炙甘草, *Glycyrrhiza uralensis* Fisch [processed root] (see Table 3)

Zhi ke 枳壳, *Citrus aurantium* L [fructus]

Zhi mu 知母, *Anemarrhena asphodeloides* Bge [rhizome]

zhong feng 中风, ‘wind stroke’

Zhong Hua Ben Cao 中华本草 (1999) book name

Zhong Hua Yi Dian 中华医典 book name: ‘Encyclopaedia of Traditional Chinese Medicine’

Zhong Yao Da Ci Dian 中药大辞典 book name: ‘Great Compendium of Chinese Medicines’

TABLE SUPP 3: Traditional properties of the most frequent herbs – channel tropism, flavour, nature

	Channel tropism[5]										Flavour[5]							Nature[5]			Ref.[1]
Herb name[1]	Heart	Liver	Spleen	Stomach	Lung	Kidney	Bladder	Gall bladder	Peri-cardium	Large intestine	Sour	Bitter	Sweet	Pungent	Salty	Bland	Neutral	Warm	Cold	Hot	Page no.
Gan cao	1		1	1	1								1				1				875
Sheng jiang[2]			1	1	1									1				1			2299
Gan jiang	1		1	1	1									1						1	2306
Dang gui	1	1	1									1	1	1				1			1349
Bai shao3		1	1								1	1							1		649
Chi shao		1	1									1							1		659
Fang feng		1	1		1		1						1	1				1			1423
Gui zhi	1				1		1						1	1				1			464
Ma huang					1		1					1		1				1			274
Bai zhu			1	1								1	1					1			1897
Chuan xiong		1						1	1					1				1			1402
Fu ling	1		1		1	1							1			1	1				189
Huai niu xi		1				1					1	1					1				405
Chuan wu	1	1	1			1						1		1						1	479
Huang qi			1		1								1					1			825
Fang ji			1		1		1					1		1					1		589
Ren shen	1		1		1	1						1	1				1				1281
Cang zhu		1	1	1								1		1				1			1889
Bai zhi			1	1	1									1				1			1332
Fu zi	1		1			1							1	1						1	587
Du huo						1	1					1		1				1			1338
Huang qin	1	1			1			1		1		1							1		1689

Chen pi			1	1	1							1		1				1			1004
Tao ren	1	1								1		1	1				1				741
Sheng di huang (gan di huang)[4]	1	1				1						1	1						1		1740
Shu di huang		1				1							1					1			1745
Rou gui	1	1	1			1							1	1						1	458
Qin jiao		1		1				1				1		1					1		1486
Huang bai						1	1			1		1							1		1044
Wei ling xian		1					1					1		1	1			1			522
Total	12	15	18	8	13	10	7	3	1	3	2	18	13	17	1	1	5	14	7	4	

Note: 1. Names are in pinyin, see glossary for scientific names; 2. Paeony is in two forms – white (bai shao) and red (chi shao); 3. Ginger is in two forms - fresh (sheng jiang) and dried (gan jiang); 4. Rehmannia is in two forms – unprocessed (sheng di huang, also called gan di huang) and steamed (shu di huang); 5. There are minor variations between sources in the channel tropism, flavour and nature of some herbs. These properties are all according to Zhong Hua Ben Cao.

Reference

1. State Administration of Traditional Chinese Medicine 'Chinese Materia Medica Committee', ed *Zhong Hua Ben Cao: Jing Xuan Ben 'Chinese Materia Medica: abridged version'*. Shanghai: Shanghai Scientific and Technical Publishers; 1998.
